# Biodegradability and Biocompatibility Study of Poly(Chitosan-*g*-lactic Acid) Scaffolds

**DOI:** 10.3390/molecules17033243

**Published:** 2012-03-14

**Authors:** Zhe Zhang, Huifei Cui

**Affiliations:** 1 Institute of Biochemical and Biotechnological Drugs, School of Pharmacy, Shandong University, Jinan 250012, China; Email: zhangzhelh@gmail.com; 2 National Glycoengineering Research Center, Shandong University, Jinan 250012, China

**Keywords:** chitosan, lactic acid, scaffold, biodegradable, biocompatible

## Abstract

A biodegradable, biocompatible poly(chitosan-*g*-lactic acid) (PCLA) scaffold was prepared and evaluated *in vitro* and *in vivo*. The PCLA scaffold was obtained by grafting lactic acid (LA) onto the amino groups on chitosan (CS) without a catalyst. The PCLA scaffolds were characterized by Fourier Transform infrared spectroscopy (FT-IR) and scanning electron microscopy (SEM). The biodegradabilty was determined by mass loss *in vitro*, and degradation *in vivo* as a function of feed ratio of LA/CS. Bone marrow mesenchymal stem cell (BMSC) culture experiments and histological examination were performed to evaluate the PCLA scaffolds’ biocompatibility. The results indicated that PCLA was promising for tissue engineering application.

## 1. Introduction

In recent years, chitosan (CS) and poly(lactic acid) (PLA) have received widespread attention as biodegradable and biocompatible materials [1,2,3]. However, CS can only be dissolved in dilute acidic solution, and as a scaffold material, CS degrades slowly, and can’t match with regeneration of tissues. Besides, CS’s mechanical properties are rather poor [4,5,6,7]. These properties limit CS’s even broader application in tissue engineering. PLA is the most widely used synthetic biodegradable macromolecule, with excellent mechanical properties and processability. Moreover, PLA’s degradation rate is adjustable. But PLA also has drawbacks [8] such as local inflammatory response and hydrophobicity. Therefore, CS and PLA can be viewed are complimentary in their characteristics as tissue engineering materials. If they could be complexed, CS can neutralize the acidic degradation products generated by PLA, while, CS’s mechanical properties could be improved, especially its brittleness resulting from its high crystallinity.

However, effectively blending CS with PLA at a highly miscible level is still a challenge due to two main difficulties: (1) CS has a high glass transition temperature [9] and will possibly start to decompose before melting, it is thus impossible to blend CS with PLA by the melting processing technique, and (2) to date, to our knowledge, no suitable co-solvent is available for CS and PLA. CS can only be dissolved in very few kinds of dilute acidic aqueous solutions, while PLA can normally only be dissolved in some organic solvents. Although several efforts have been dedicated to blending PLA with CS, unfortunately, few of them were successful and obvious phase separation was observed in their blends [10,11,12,13]. In this research, PCLA was obtained by grafting lactic acid onto the amino groups in CS by vacuum freezer drying and vacuum reaction without a catalyst. Its structure was characterized by FT-IR.

BMSCs [14,15,16] are undifferentiated multipotent cells which exist in various human tissues. They could be regarded as a repository of reparative cells in the human body that are capable on mobilizing, proliferating and differentiating into the appropriate cell type in response to certain signals [17,18,19,20]. Our previous study has shown that some of the co-grated BMSCs have differentiated into nerve-like cells in implanted chitosan conduits which might be helpful for rat sciatic function repair. Therefore, in this study, BMSCs were used for cytocompatibility evaluation. Histological examination was also performed to investigate PCLA’s biocompatibility.

## 2. Results and Discussion

PCLA materials were successfully fabricated by grafting lactic acid onto the amino groups in CS by a vacuum reaction. The freshly prepared PCLA scaffolds were buff and brittle. When exposed to air, they easily absorb moisture and become sticky and soft. 

### 2.1. FTIR Analysis

**Figure 1:**
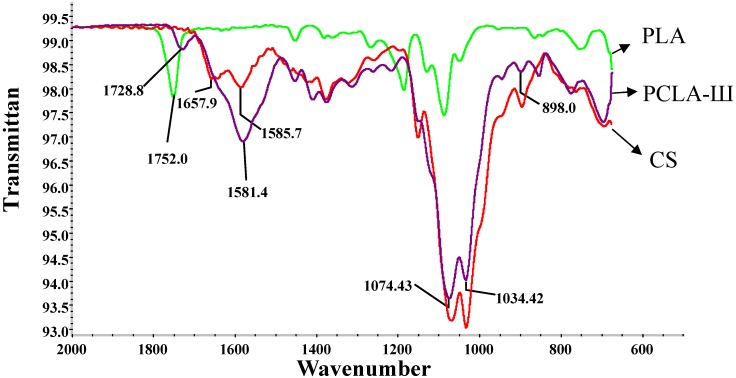
IR spectra of CS, PLA and PCLA-III (LA: CS = 4:1, w/w) copolymer.

PCLA-III was taken as a representative of the copolymers prepared. The IR spectrum of CS (Figure 1) shows peaks assigned to the saccharine structure at 898.0, 1,034.42 and 1,074.43 cm^−1^ and a strong characteristic amino peak at around 1,585.7 cm^−1^. The shoulder peak at 1,657.9 cm^−1^ is attributed to the amide II band of the amino group. Compared to the IR spectrum of CS, the PCLA copolymer has a new peak present around 1,728.8 cm^−1^ (Figure 1) corresponding to the ester groups of OLA existing as side chains. An obvious shift to a lower wavenumber in comparison with PLA can be observed and is attributed to the formation of hydrogen bonds between the ester groups of OLA and amino or hydroxyl groups of CS. The new absorption band at around 1,581.4 cm^−1^ is a result of the overlapping of the peaks from the amide I bands and the amino groups of CS with the peaks from the salt links that conjoin CS with OLA. The shift of the amino groups’ absorption from 1,585.7 cm^−1^ of CS to 1,581.4 cm^−1^ of the PCLA copolymer (Figure 1) means that OLA is linked with CS through both amide bonding and static electron interactions.

From the IR spectra we can see that PCLA had two typical bands which were amide I: N-acylamide and amide II: amino group [21], the peak strength corresponding to the amide I band of CS was enhanced, indicating the attachment of PLA side chains. Compared with the spectrum of CS, PCLA had a clear new peak, which should be assigned to the ester or carboxylic groups in PLA side chains [22]. The results obtained from the IR spectra suggest that PLA side chains have been successfully grafted onto the chitosan main chains.

### 2.2. SEM and Porosity

**Figure 2:**
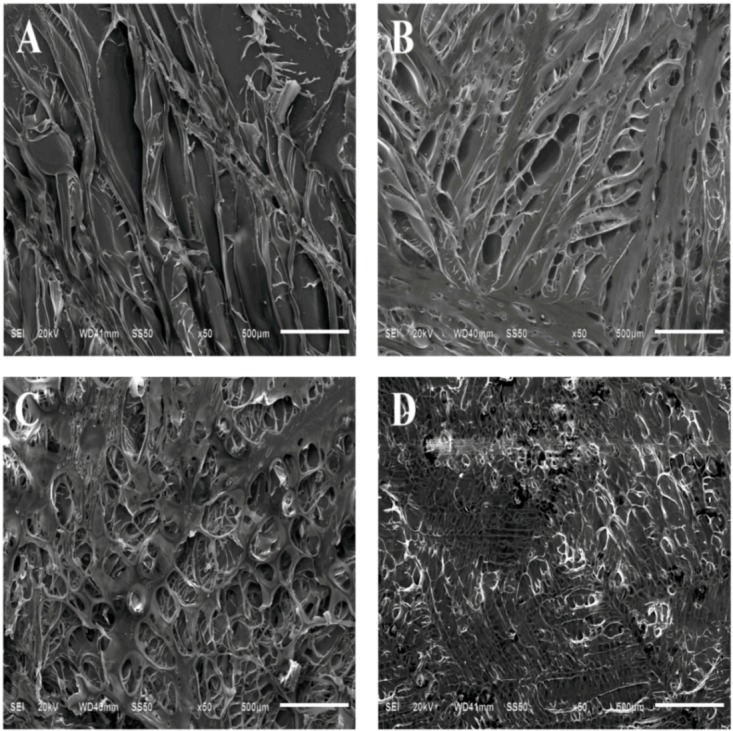
SEM images of the outer surfaces of (**A**) CS; (**B**) PCLA-Ι (LA: CS = 2:1, w/w); (**C**) PCLA-Ⅱ (LA: CS = 3:1, w/w); (**D**) PCLA-Ⅲ (LA: CS = 4:1, w/w).

As shown in Figure 2, both CS and PCLA scaffolds exhibited three-dimensionally interconnected porous structures with a pore size around 100–500 µm. The pore size of the PCLA scaffolds decreased with increasing LA/CS feed ratio. The CS has a porosity of 62.3% and pore size of 500 µm, and the PCLA-III has a porosity of 34.37% and pore size of 100 µm. This was supported by the porosity values listed in Table 1.

The scaffolds prepared were porous and the thickness of the composite scaffold was approximately 1 mm. PCLA scaffolds’ porosity decreased as the LA/CS ratio increased from 2 to 4. Qu [19] also obtained similar results. When the LA/CS ratio increases, the copolymer will have longer side chain length, consequently, a more firmly physical cross-linked copolymer with decreased porosity will be obtained.

For repair applications, ideal scaffolds should have appropriate pore structures, pore sizes and porosity to ensure a biological environment conducive to cell attachment, proliferation and flow transport of nutrients and metabolic waste. The morphology shown in Figure 2 indicated that the porosity and pore size of the PCLA scaffolds decreased along with increase in LA/CS feed ratio. 

**Table 1:** Basic parameters of copolymers (mean ± SD, n = 6).

Copolymer Samples	CS	PCLA-Ι	PCLA-ΙΙ	PCLA-ΙΙΙ	PLA
LA/CS (wt/wt)	-	2	3	4	-
[COOH]/[NH_2_] (mole ratio)	-	3.27	4.91	6.54	-
Porosity	62.03 ± 0.07	46.97 ± 0.06 ^#^	41.30 ± 0.05 *	34.37 ± 0.06 *	0

^a^ porosity: measured by ethanol volume displacement method [17]; Statistically significant compared with CS group: * *p *≤ 0.05, ^#^* p *≤ 0.01.

### 2.3. Mass Loss *in Vitro*

**Figure 3:**
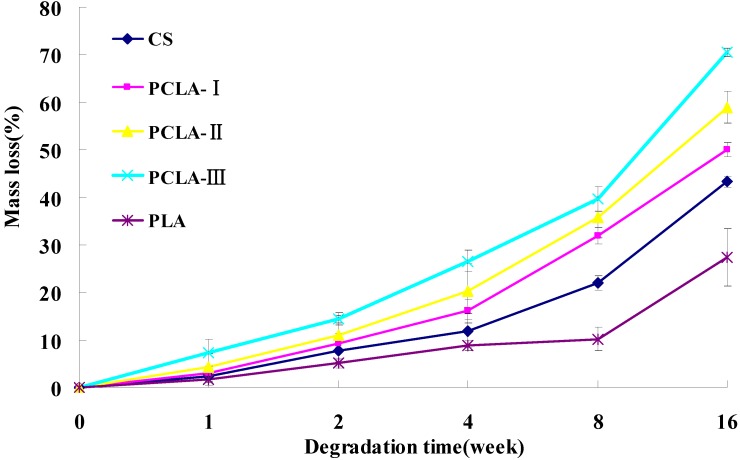
Degradation of PCLA materials by lysozyme (4 mg/mL) at pH = 7.4 and 37 °C (mean ± SD, n = 3).

Figure 3 shows the mass loss of PLCA scaffolds after incubation in PBS with addition of 4 mg/mL lysozyme solutions for various periods. The mass loss trend of CS and PCLA was analogous, with slow degradation in the first week and then a dramatic mass loss after 2 weeks. The PCLA-III degraded much faster than others, losing 70.5% of the weight compared with CS (43.3%) at the 16 week point. However, the mass loss of PLA remained relatively slow throughout degradation with a mass loss 27.4% by the end.

**Figure 4:**
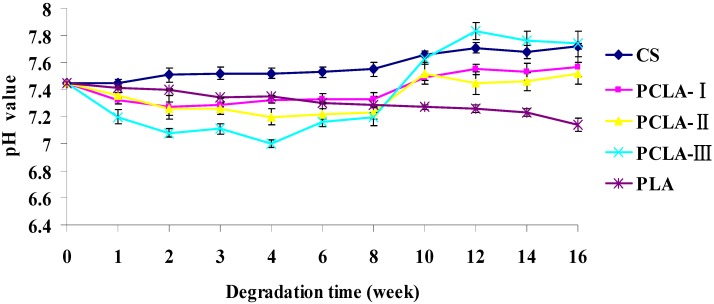
The pH value in PBS by sodium azide (0.01 wt %) at 37 °C for up to 16 weeks(mean ± SD, n = 3).

Varum *et al*. [23] showed that CS was degraded by lysozyme in animals, so the copolymers were incubated in lysozyme solution and monitored the mass loss after enzymatic hydrolysis, which was one of the most concerning characteristics of the copolymers. All the PCLA copolymers lost mass quickly because of the hydrolysis of grafted lactic acid which can be proven by the pH change of the PCLA (Figure 4). The grafted lactic acid were easily hydrolyzed from the backbone of CS and hydrolyzed faster as the LA/CS increased which led to a faster mass loss and pH drop of the PCLA composites in the first 8 weeks. From the 9th to 16th week, the lactic acid hydrolyzed slower. The hydrolyzed lactic acid promoted the degradation of CS which led to the rising pH of the PCLA and showed pH wave character, meanwhile, the mass loss ratio became faster. However, PLA showed very little mass loss and pH change, which was different from earlier research, perhaps because the PLA we used was the molecular weight of 100,000 which was higher than others but was used for tissue engineering widely.

### 2.4. pH Fluctuation

The pH fluctuation during the incubation is presented in Figure 4. The pH value of degradation media for incubating CS increased gradually through the whole degradation period and reached around 7.72 at the end of 16 weeks of incubation. On the other hand, the pH value of the media for incubating PLA decreased at a slow rate, and reached around 7.14 at the end of 16 weeks of incubation. Because the PLA used in our research has a molecular weight of 100,000 and it degraded slowly, the pH of degradation media for incubating PCLA-I and PCLA-II decreased gradually through the 1st–4th week and reached around 7.32 and 7.2 at the end of the 4th week of incubation, but after the 4th week, the pH began to waver. They showed an upward trend from the 8th week and reached 7.57 and 7.52 at the end of 16-week incubation, respectively. However, the pH value of PCLA-Ⅲ decreased significantly through the 1–4 weeks and reached around 7.0 at the end of the 4th week, then the pH increased gradually through the 4th–12th week reaching the highest pH at 7.83. After that it began to go down to 7.74 at the end of 16 weeks of incubation.

### 2.5. Mass Loss *in Vivo*

**Figure 5:**
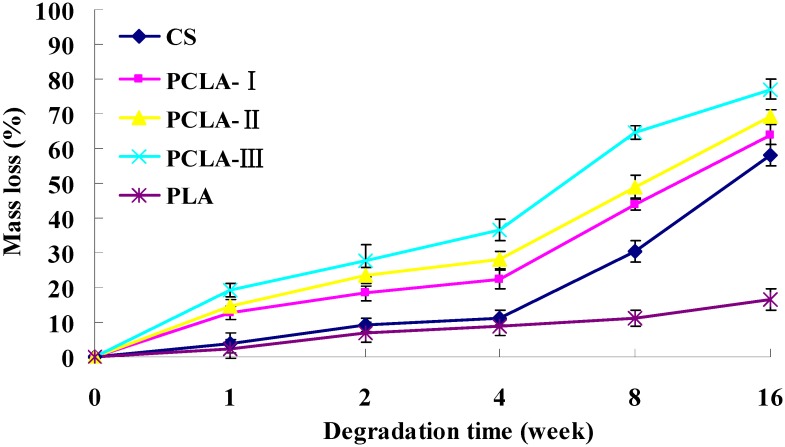
*In vivo* biodegradation data for CS, PLA and PCLA scaffolds subcutaneously implanted into the backs of Kunming mice for up to 16 weeks (mean ± SD, n = 6).

After implantation, the morphology of the implanted copolymers changed progressively as time went by and finally disintegrated. Figure 5 showed the mass loss of the copolymer *in vivo*. The PLA, which lost 16.67% by the end of 16 weeks had the lowest degradation rates among the five groups. The degradation trend of the PCLA copolymers was similar to CS but faster. They degraded slowly during the 1st week and experienced much slower mass loss during the 2nd–4th week, after that a dramatic mass loss appeared during the 4th–8th week. The PCLA-III had the fastest degradation rates and reached 77% of mass loss at the end of 16 weeks.

*In vivo* mass loss was calculated by weighing the mass of the scaffold prior to implantation and then post-implantation after a tissue digestion protocol. It was noted that the CS and PCLA scaffolds degraded more quickly *in vivo* (Figure 5) than *in vitro* (Figure 3), but the PLA scaffolds degraded slowly *in vivo* than *in vitro*, which was opposite to Lu’s research [24]. This may be caused by PLA’s different molecular weight and specimen structure. In the present study, PLA was higher and non-porous which was unsuitable for its hydrolysis, moreover, water *in vivo* was less than *in vitro*. Other studies have also shown similar results for CS [25,26].

### 2.6. Cytocompatible Evaluation

**Figure 6:**
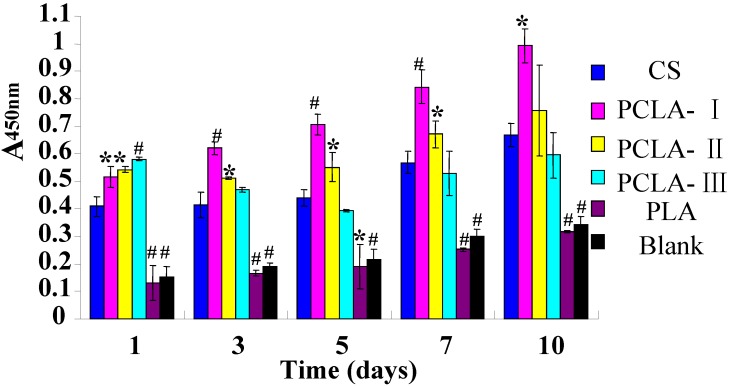
The growth of BMSCs on materials for 1, 3, 5, 7, 10 days cultivation, viability was determined by the MTT assay and analysis by the one-way ANOVA (mean ± SD, n = 3; * *p* ≤ 0.05 compared with CS and **^#^** *p* ≤ 0.01 compared with CS).

Figure 6 illustrates the cell proliferation on the different scaffolds. The cell activity represented by the cell number was found to increase with culture time, indicating that BMSCs could attach and proliferate on the surface of the scaffolds. For the PLA scaffold, there is no evident increase in the cell number at all time intervals, indicating that PLA does not promote cell growth and proliferation. Compared with that of CS, cell numbers on PCLA scaffolds increased significantly during the culture time. The cell growth rate in the PCLA scaffolds was related to the feed ratio of LA/CS. The cell growth rate on the copolymer scaffolds was faster than CS obviously and increased with the rising of LA/CS feed ratio. However, from the third day, the cell number on CS scaffold exceeded that on PCLA-III scaffold, but less than that of PCLA-I and PCLA-II scaffolds.

When fibroblasts were cultured on PCLA composites, Wan *et al.* [14] showed that there were no substantial differences in the viability, density and distribution of fibroblasts between PCLA fibrous scaffolds and pure CS fibrous scaffolds. However, it was the different for BMSCs (Figure 6). The PCLA copolymers’ spatial structure may be advantageous for cell adherence and growth with the rising LA/CS ratio. Therefore, the cell growth rate on the PCLA scaffolds increased with LA/CS rise on the first day. While the acid-effect also increased with the ratio LA/CS rising which led to the cell growth rate decreasing later on the PCLA scaffolds with rising LA/CS value. The PLA group had very low cell number at all time intervals due to its hydrophobic surface which was disadvantageous for cell adherence.

### 2.7. Histocompatibility

**Figure 7:**
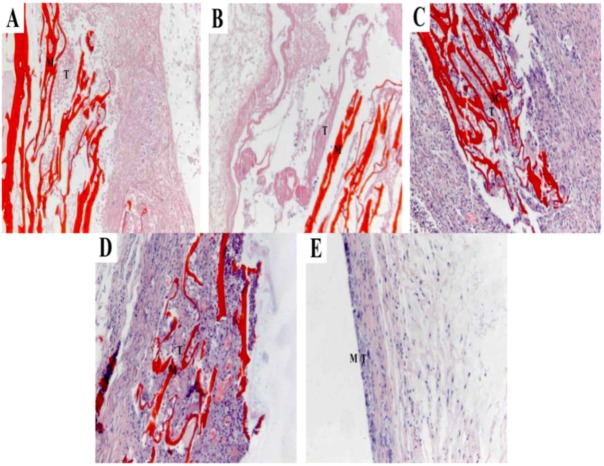
Histological sections of CS, PCLA and PLA scaffolds after 1 week of implantation (**A**) CS; (**B**) PCLA-Ι; (**C**) PCLA-ΙΙ; (**D**) PCLA-ΙΙΙ; (**E**) PLA.M stand for materials and T stand for tissues (100×).

All rats underwent implantation surgeies survived during the 8 postoperative weeks. Some of the implanted sites of the rats showed infection and inflammation after the operation. Figure 7 shows H&E histological staining of the CS, PCLA and PLA scaffolds after implantation 1 week. 

**Figure 8:**
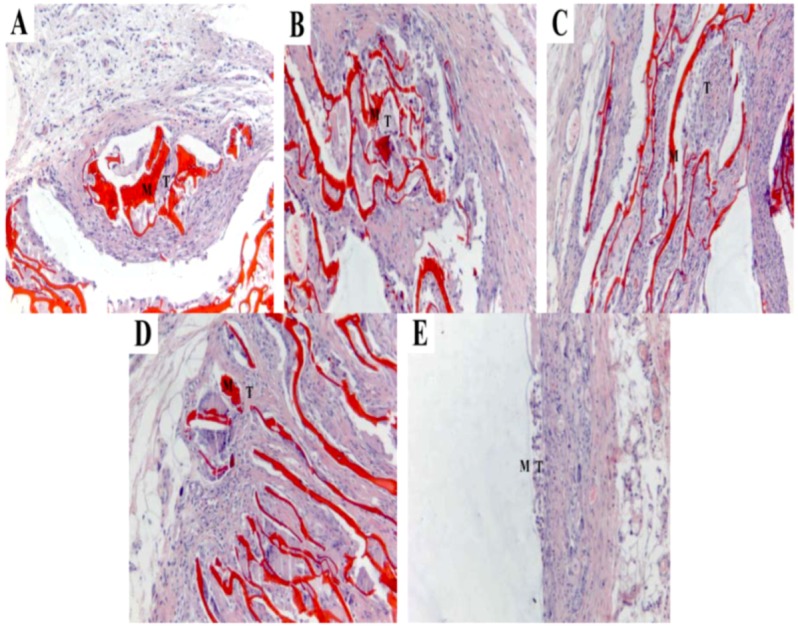
Histological sections of CS, PCLA and PLA scaffolds after 4 weeks of implantation (**A**) CS; (**B**) PCLA-Ι; (**C**) PCLA-ΙΙ; (**D**) PCLA-ΙΙΙ; (**E**) PLA.M stand for materials and T stand for tissues (100×).

Both tissue response and degradation within and surrounding the scaffold could be seen. Inflammatory cells were present around the CS and PCLA-I and macrophages were around the materials of PCLA-II and PCLA-ΙΙΙ. However, the tissue could not adhere to the surface of the PLA for its hydrophobicity. At 4 weeks of implantation, as shown in Figure 8, all the scaffolds in the CS, PCLA and PLA groups were encapsulated by fibrous collagen. The interface between scaffold and tissue was not clearly visible except the PLA. What’s more, the tissue around the PLA can be easily removed. The inflammation around the materials disappeared and the tissue began to embed into the materials except the PLA.

**Figure 9:**
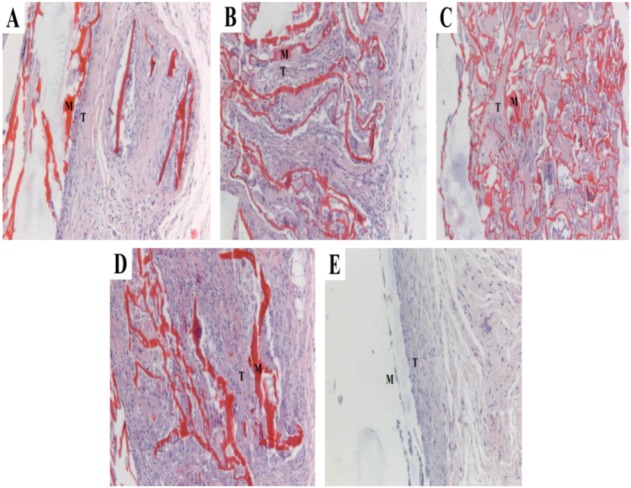
Histological sections of CS, PCLA and PLA scaffolds after 8 weeks of implantation (**A**) CS; (**B**) PCLA-Ι; (**C**) PCLA-ΙΙ; (**D**) PCLA-ΙΙΙ; (**E**) PLA.M stand for materials and T stand for tissues (100×).

At 8 weeks of implantation, as shown in Figure 9, the amount of macrophages around the CS and PCLA scaffolds was reduced and scaffolds were encapsulated by fibrous collagen. The surface of PLA began to decompose and the interface between scaffold and tissue was not clearly visible.

In the *in vivo* setting, there were both phagocytosis and hydrolytic modes of degradation acting simultaneously, as well as the lactic acid contributing to the breakdown of the material. It seems that macrophages play an important role in the phagocytosis of the material and its degradation *in vivo*. Phagocytic behavior seems to be influenced by the surface properties of scaffolds [27]. The surface of PCLA was more suitable for BMSCs to adhere, so it is also more suitable for macrophages to adhere. What’s more, the hydrolyzed lactic acid may cause acidic inflammation (Figure 7), which would contribute to macrophage stimulation. Moreover, a previous study on CS reported its preferential degradation in acidic conditions [28]. The inflammatory site with acidic pH represents an adequate site for CS degradation acceleration in parallel with macrophage phagocytosis leading to copolymer resorption upon implantation [29,30]. PCLA scaffolds used in this study were prepared from the same type of CS with a specific molecular weight and degree of deacetylation. It may be important to investigate other types of PCLA scaffolds since their composition seems to be a determinant factor during the degradation process. Studies on biodegradable copolymers reported that molecular weight and copolymer composition were important and may influence the biodegradation rate [31,32,33]. 

Histological analysis was carried out in order to evaluate inflammatory cell infiltration during the acute inflammation stage (1 week time point) and long-term tissue formation while the materials decomposed (up to 8 weeks). It was very hard to do a biopsy for the CS and PLA. Because CS was so brittle to shred and the PLA was very hard to cut, while the PCLA behaved well. At the early 1 week time point, both CS scaffolds showed signs of cell infiltration into the pores; so did with PCLA scaffolds which was possibly due to the easily hydrolyzed lactic acid from the grafted lactic acid. Caution is indicated when interpreting the *in vivo* results as no quantitative analysis has been performed and as other variables which include biodegradability, surface characteristics, size, and chemical composition can also significantly influence local histological response to an implant [34]. These factors must be taken into account when attempting to compare reactivity among different materials [35]. However, as the time went by, the hydrolyzed lactic acid accelerated the macrophage stimulation and the scaffolds degradation. The basic degradation products of the CS buffered the acid-effect and the inflammation of PCLA relived, so there were few inflammatory cells or macrophage present in the PCLA scaffolds at the 8th week after implantation, but the PLA scaffolds decomposed slowly and the embedded tissue could be seen at 8 weeks. What’s worse, the tissue could be removed easily from the surface of PLA due to its hydrophobicity. The histocompatibility and hydrophilicity are very important for tissue engineering, and PCLA seems an ideal biomaterial for tissue repair.

## 3. Experimental

### 3.1. Materials

CS was purchased from Jinan Haidebei Marine Bioengineering Company (China) and was prepared from Alaska deep-sea crab shell. The molecular weight of CS used are 5 × 10^5^ Da (determined by Gel Permeation Chromatography, GE Healthcare, Piscataway, NJ, USA), the deacetylation degrees are 85%, respectively (determined by acid-base titration). All other chemicals were of analytical grade and used without further purification: lactic acid (LA, 88%), methanol, sodium hydroxide pellets, acetic acid, ethanol.

### 3.2. Synthesis of PCLA

PCLA was synthesized using a method described elsewhere [14,15]. In brief, CS powder was dissolved in a aqueous solution of lactic acid to prepare a 2.5 wt % solution. The solution was cast onto a plastic dish as a very thin liquid membrane and then put immediately into a refrigerator to freeze at −80 °C over night after being lyophilized in a vacuum freeze-drying and then dried in the vacuum drying oven for 8 h at 80 °C to dehydrate the copolymer salt and promote the formation of corresponding amide linkages. The unreacted lactic acid and oligo(lactic acid) (OLA) were removed by extracting the membrane samples with methanol until the OLA can’t be detected by the UV-Vis spectrophotometer.

### 3.3. Characterization of Scaffolds

The infrared (IR) spectra were recorded on a Nicolet NEXUS 470 FT-IR spectrometer with a resolution of 8 cm^−1^, 200 scans, in transmission mode. The samples were prepared by OMNI-Sampler.

The surface morphologies of the samples were observed under scanning electron microscope (SEM) (Hitachi, SU-70, Pleasanton, CA, USA). The porosity of the PCLA scaffolds were measured by liquid displacement, as described elsewhere [17]. Ethanol was used in procedure because it readily penetrated the pores of the matrices and did not induce shrinkage or swelling of the material.

The weight of dry PCLA scaffold was W_d_, the weight of pycnometer full of ethanol was W_1_. Put the dry PCLA scaffold immersed in a pycnometer full of ethanol for 24 h until no air bubbles were observed emerging from the PCLA scaffold. The total weight of ethanol and the ethanol-impregnated PCLA scaffold was recorded as W_2_. The ethanol-impregnated PCLA scaffold was removed from the pycnometer and the weight of residual ethanol and pycnometer was recorded as W_3_. ρ stand for the density of the ethanol. The porosity (ε) was calculated using the followed equations:

The volume of the PCLA scaffold was:


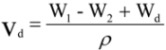
(1)

The volume of the pores in the PCLA scaffold was:


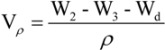
(2)

and the porosity of the foam (ε) was obtained by:


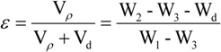
(3)

### 3.4. Mass Loss *in Vitro*

The degradation of PCLA scaffold was evaluated by putting dry PCLA scaffold (1 × 1 cm^2^) in 4 mg lysozyme/mL solution in PBS (pH = 7.4) incubated at 37 °C [36]. The hydrolysis solution was kept fresh by replacement of the lysozyme solution once a week. At determined time intervals, samples were removed from the degradation solution, rinsed, and vacuum dried. The mass loss was measured as the ratio of the weight after enzymatic hydrolysis to the PCLA scaffold.

### 3.5. pH Change

A PBS (pH = 7.4) solution was first prepared by dissolving PBS powder in deionized water [37]. The PCLA scaffolds were weighed and then immersed in the PBS solution with 0.01 wt % of sodium azide in sealed glass tube. The PCLA scaffolds were incubated at 37 °C for various durations without refreshing media. At the end of predetermined incubation intervals, the medium of three samples per group were collected for the pH measurement using a pH meter (PB-10, Sartorius Instruments, Shanghai, China).

### 3.6. Mass Loss *in Vitro*

Seventy-five Kunming mice (6 weeks, weighing 20 ± 2 g) were used for testing the biodegradability of scaffolds. Animals were purchased from the Experimental Animal Center of Shandong University, and treated in accordance to the ‘NIH Guidelines for the Care and Use of Laboratory Animals’. The mice were randomly divided into five groups (5 × 5 × 3 = 75), for implanting the five kinds of scaffolds mentioned above, respectively. After being anesthetized by an intravenous injection of sodium pentobarbital (30 mg/kg body weight), one piece of scaffold was interposed subcutaneously (two pieces of scaffolds for each mouse). Incisions were closed using 4–0 sutures. Animals were routinely housed following surgical procedures. 

After 1, 2, 4, 8 and 16 weeks, scaffolds were taken out from the mice (three mice were randomly selected from a group each time) by stripping the adherent tissue carefully. Explanted scaffolds was washed three times in Dulbecco’s phosphate-buffered saline(D-PBS; PBS without Ca^2+^ or Mg^2+^, pH = 7.2) and soaked in 2.5 mg/mL protease A in PBS solution (with Ca^2+^, Mg^2+^) for 2–4 h at 37 °C. The protease A solution was replaced with 1 mL of 2.5 mg/mL collagenase [38] in PBS solution (with Ca^2+^, Mg^2+^) and left to incubate overnight at 37 °C. If the tissue was not totally disintegrated, the collagenase solution was replaced with fresh collagenase solution (2.5 mg/mL) and incubated for an additional 48 h. Scaffolds were then washed three times in wash buffer (50 mM Tris HCl,10 mM CaCl_2_, pH = 8.0) and sonicated for 10 min between each wash. Scaffolds were then soaked in wash buffer with 100 mg/mL proteinase K [39] and left to incubate overnight at 37 °C. The next day, the proteinase K was removed and the scaffolds were washed three times in wash buffer while sonicating for 15 min each time. Scaffolds were then sonicated for a total of 3 h in lysis buffer (100 mM Tris HCl, 100 mM NaCl, 25 mM EDTA, 0.1% SDS, pH = 8.0) replacing buffer after each hour of sonicating. This was followed by three 30-min sonications in sterile filtered, distilled water. Scaffolds were then dehydrated in increasing concentrations of ethanol (30%, 50%, 70%, 90%, 95%, and 100% for 1 h each) and left to air-dry for three days in a fume hood prior to weighing using an analytical balance (±0.0001 g) and the percent mass loss calculated.

### 3.7. Cytocompatibility Evaluation

For isolation of rat BMSCs, tibias and femurs were dissected from adult Wistar rats (200–250 g, Animals were purchased from the Experimental Animal Center of Shandong University, and treated in accordance to the ‘NIH Guidelines for the Care and Use of Laboratory Animals’). After the ends of the bones were cut, the marrow was extruded with 10 mL D-Hank’s solution and resuspended in DMEM solution. About 1 × 10^7^ marrow cells were plated on 25 cm^2^ plastic flask in DMEM, supplemented with 10% FBS, 2 mM L-glutamine, 100 U/mL penicillin, and 100 mg/mL streptomycin. BMSCs were isolated by short-term adherence to plastic as described previously [18,19]. After 24 h, nonadherent cells were discarded by replacing the medium and adherent cells were cultured. The medium was added and replaced every 3 days for about 14 days. When the cells grew to confluent, they were harvested with 0.25% trypsin and 1 mM EDTA for 5 min at 37 °C, replated on 25 cm^2^ plastic flask, the cells were detached and serially subcultured. Only cells at passage two (P2) were used in this study.

BMSCs were diluted to a density of 1 × 10^5^ cells/mL and were added to 24-well tissue culture clusters embedded with PCLA, CS or PLA membranes. The clusters were incubated in DMEM medium containing 10% fetal bovine serum and 100 U/mL penicillin and streptomycin, in a 37 °C humidified incubator with 5% CO_2_. Half of the culture medium was replaced every 2 days. After 1, 3, 5, 7 and 10 days incubation, the viability of every group was assessed using the MTT assay [20]. The OD of all wells was measured photometrically at 450 nm with a Multiskan MK3 reader (Thermo Lab Systems, Ottawa, Canada). A one-way ANOVA was used to compare the means of different groups, and statistical significance was accepted at the 0.05 confidence level. 

### 3.8. Histocompatibility

#### 3.8.1. Rat *in Vitro* Protocol

Wistar rats (3 months old, 200–250 g, all male) were purchased from the Experimental Animal Center of Shandong University, and cared for out in accordance to the ‘NIH Guidelines for the Care and Use of Laboratory Animals’). The rats were randomly divided into five groups (5 × 4 × 3 = 60), for implanting the five kinds of scaffolds mentioned above, respectively. After being anesthetized by an intravenous injection of sodium pentobarbital (30 mg/kg body weight), one piece of scaffold was interposed subcutaneously (two pieces of scaffolds for each rat). Incisions were closed using 4–0 sutures. Animals were routinely housed following surgical procedures for 1, 2, 4, and 8 weeks. After being anesthetized by an peritoneal injection of sodium pentobarbital (30 mg/kg body weight), at each given time point, the rats were initially anesthetized with 5% isofluorane and then maintained at 2.5% isofluorane as samples were explanted with a small amount of surrounding tissue being removed. Post-explantation, the rats were sacrificed while anesthetized by euthanasia with carbon dioxide followed by cervical dislocation. 

#### 3.8.2. Histological Staining

CS, PLA and PCLA scaffolds explanted after 1, 2, 4 and 8 weeks were washed in cold D-PBS (pH = 7.4), fixed in 4% paraformaldehyde, and dehydrated using 15% and 30% sucrose in D-PBS (pH = 7.4) solutions. The disks were then paraffin embedded, sliced into 20 mm thick specimens, and stained using hematoxylin and eosin, staining kits from Sigma-Aldrich.

## 4. Conclusions

In this study, through grafting LA onto the amino groups in CS, a novel biodegradable and biocompatibility PCLA was synthesized and characterized by FTIR and SEM. The porosity and pore size of the PCLA was suitable for tissue engineering. The mass loss in *vivo* was faster than *in vitro* and pH value fluctuated during the *in vitro* incubation. BMSCs grew well in the PCLA materials and *in vivo* histological examination on the PCLA scaffolds showed that the PCLA has the better histocompatibility than CS or PLA. The results suggested that PCLA has promising potential as a biomaterial in tissue engineering.
